# Supplementary material for: Early individualized risk prediction using clinical data for children during the febrile phase of dengue in outpatient settings in Vietnam and Thailand
Source: PLOS Digit Health. 2026 Feb 9;5(2):e0001171. doi: 10.1371/journal.pdig.0001171 (PMC12885294; doi:10.1371/journal.pdig.0001171)
Supplement: S1 Fig — (DOCX) [file pdig.0001171.s001.docx]

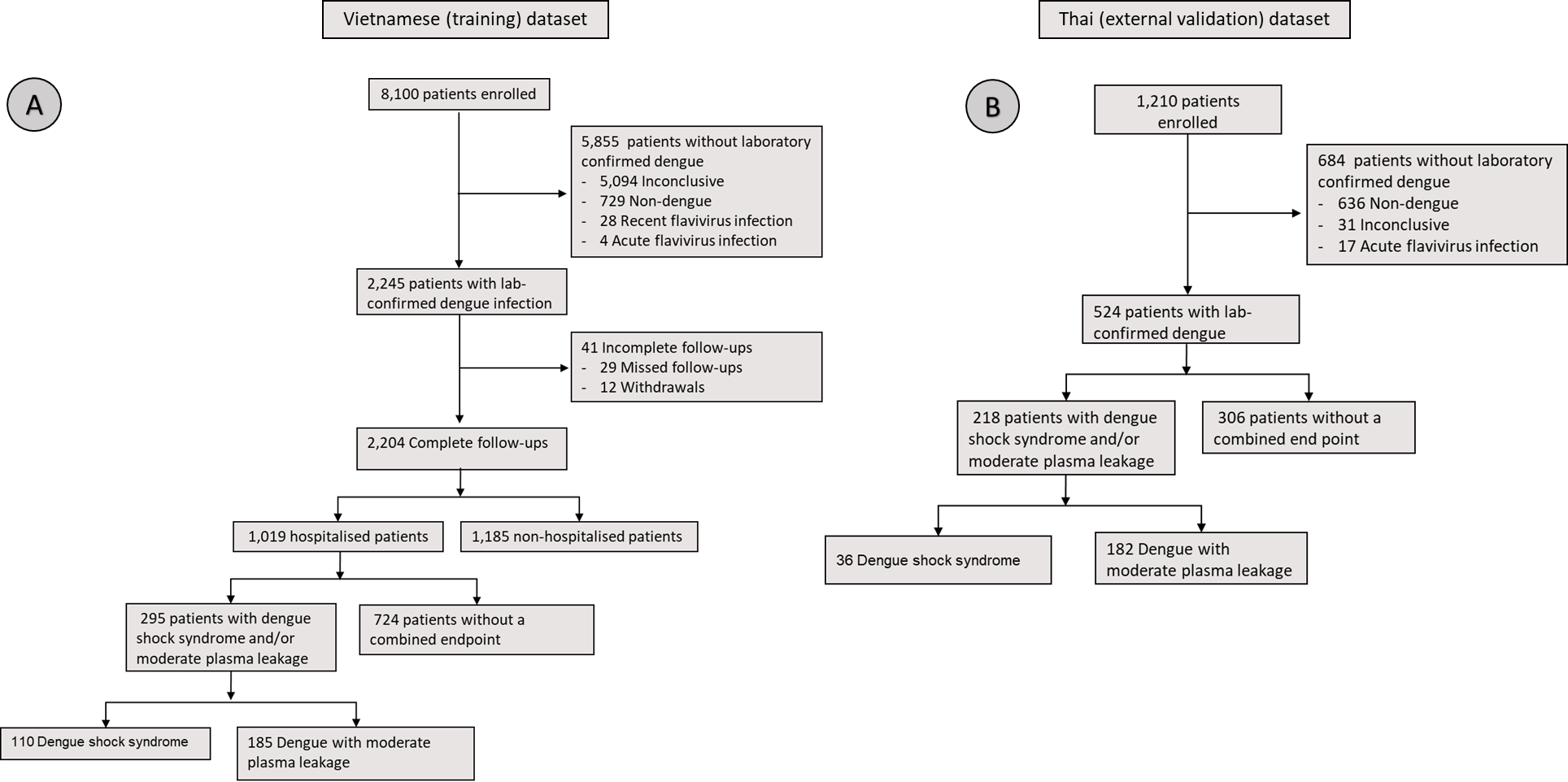


**S1 Fig**. Flow chart describing patients’ outcomes in the Vietnamese training (A) and Thai external validation datasets (B).
