# Supplementary material for: Early individualized risk prediction using clinical data for children during the febrile phase of dengue in outpatient settings in Vietnam and Thailand
Source: PLOS Digit Health. 2026 Feb 9;5(2):e0001171. doi: 10.1371/journal.pdig.0001171 (PMC12885294; doi:10.1371/journal.pdig.0001171)
Supplement: S2 Fig — The vertical and horizontal dotted lines reflect no relative difference in standard deviation and no difference in means of predicted logarithmic odds between the training and validation sets. LR: models with logistic regression and lasso selection; RF: models with random forest; XGB: models with extreme gradience boosted tree; SVM: models with support vector machine; ANN: models with artificial neural networks with 2 hidden layers. (DOCX) [file pdig.0001171.s002.docx]

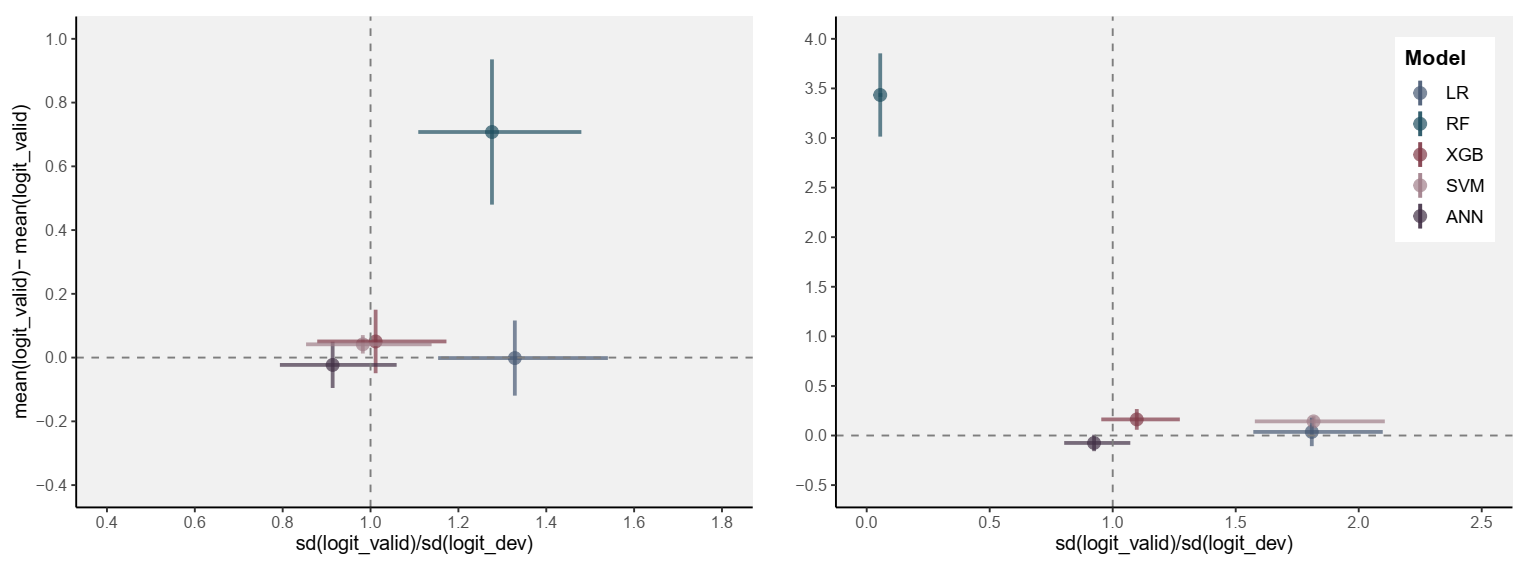


**S Fig 2**. Results of difference in means with 95% CI (the y axis) and relative difference with 95% CI in standard deviation (the x axis) of predicted logarithmic odds on the training (Left) and validation sets (Right). The vertical and horizontal dotted lines reflect no relative difference in standard deviation and no difference in means of predicted logarithmic odds between the training and validation sets. LR: models with logistic regression and lasso selection; RF: models with random forest; XGB: models with extreme gradience boosted tree; SVM: models with support vector machine; ANN: models with artificial neural networks with 2 hidden layers.
