# Supplementary material for: Early individualized risk prediction using clinical data for children during the febrile phase of dengue in outpatient settings in Vietnam and Thailand
Source: PLOS Digit Health. 2026 Feb 9;5(2):e0001171. doi: 10.1371/journal.pdig.0001171 (PMC12885294; doi:10.1371/journal.pdig.0001171)
Supplement: S5 Fig — (DOCX) [file pdig.0001171.s003.docx]

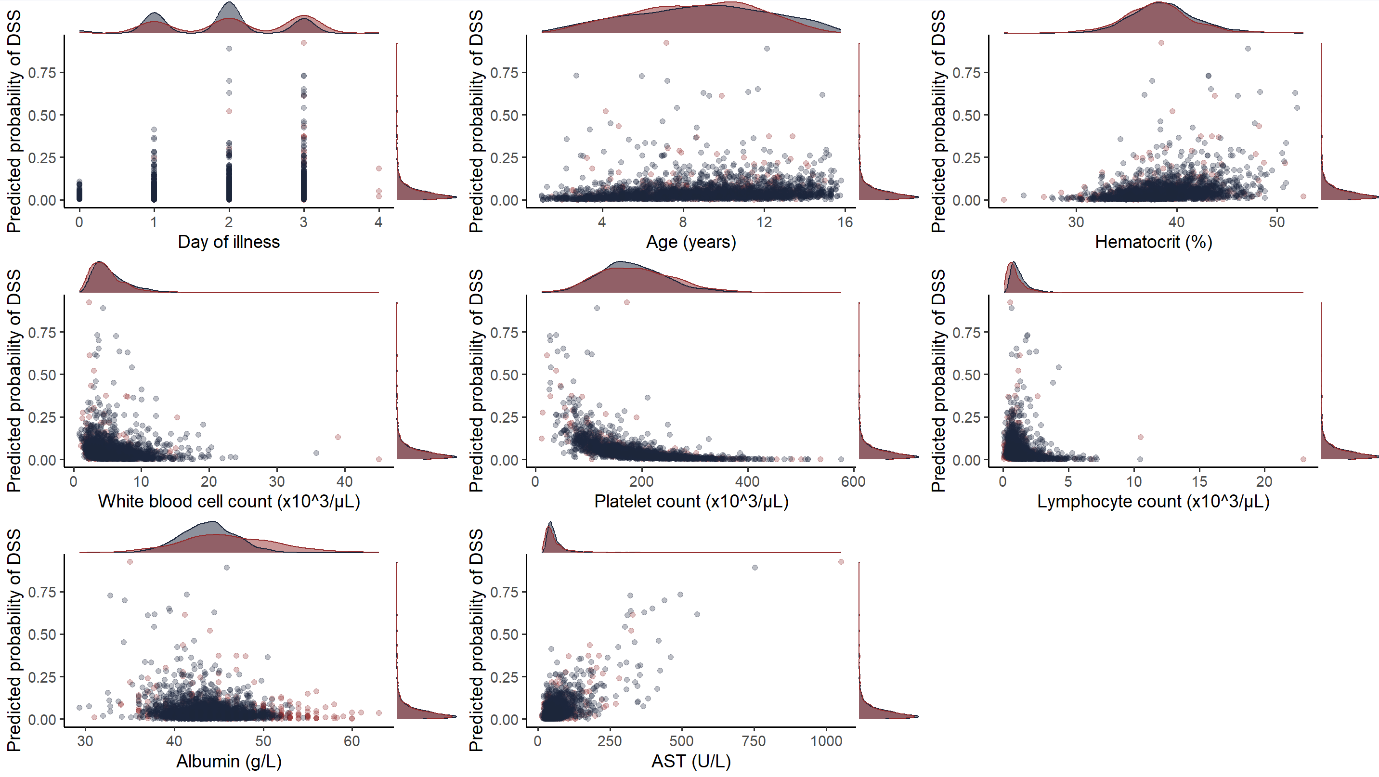


S5 Fig. Predicted probability of dengue shock syndrome (DSS) by day of illness, age, hematocrit, white blood cell count, platelet count, lymphocyte count, albumin, and AST, stratified by country (Thailand vs. Vietnam)
