# Supplementary material for: Early individualized risk prediction using clinical data for children during the febrile phase of dengue in outpatient settings in Vietnam and Thailand
Source: PLOS Digit Health. 2026 Feb 9;5(2):e0001171. doi: 10.1371/journal.pdig.0001171 (PMC12885294; doi:10.1371/journal.pdig.0001171)
Supplement: S6 Fig — (DOCX) [file pdig.0001171.s004.docx]

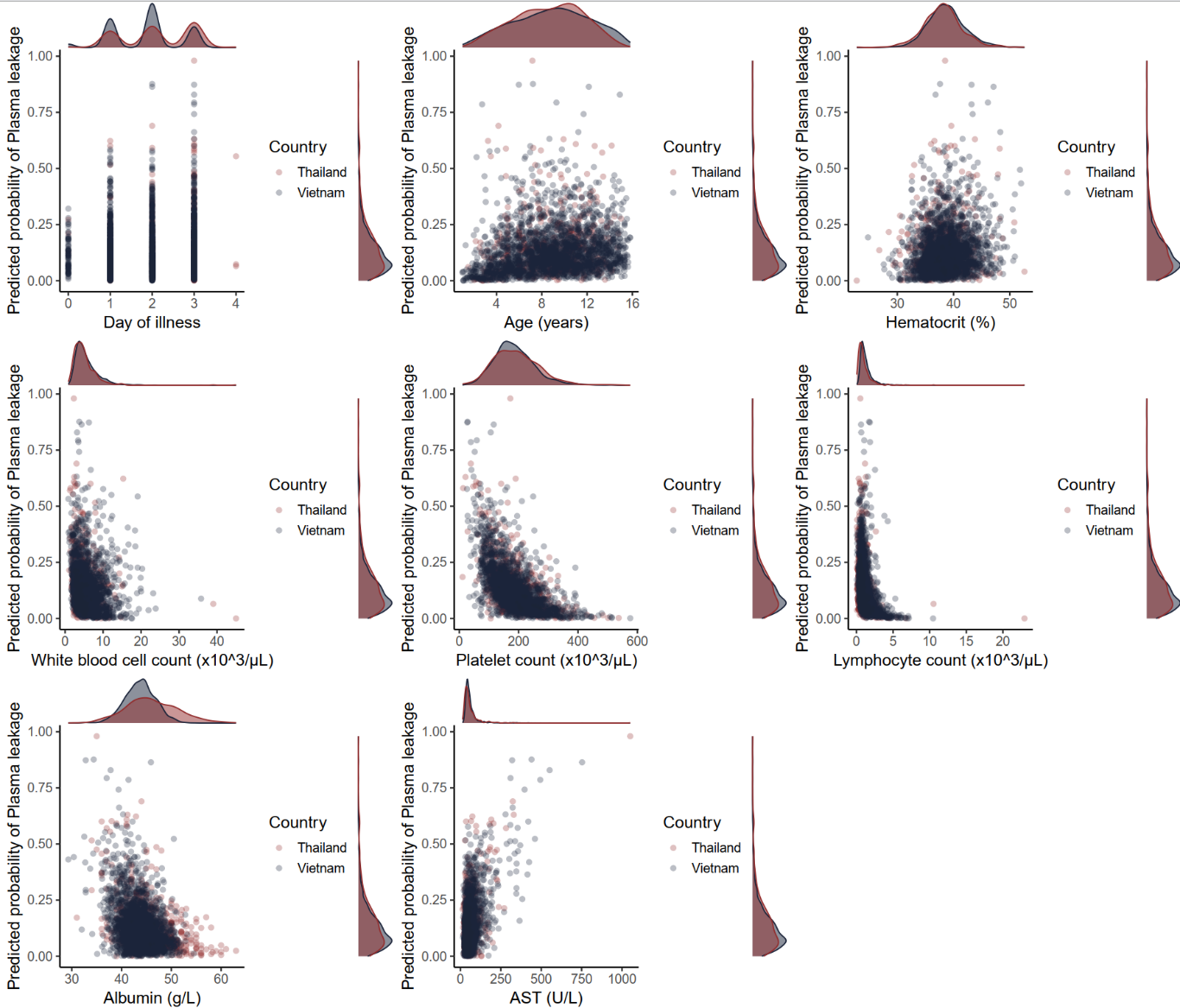


S6 Fig. Predicted probability of plasma leakage by day of illness, age, hematocrit, white blood cell count, platelet count, lymphocyte count, albumin, and AST, stratified by country (Thailand vs. Vietnam).
