# Supplementary material for: Early individualized risk prediction using clinical data for children during the febrile phase of dengue in outpatient settings in Vietnam and Thailand
Source: PLOS Digit Health. 2026 Feb 9;5(2):e0001171. doi: 10.1371/journal.pdig.0001171 (PMC12885294; doi:10.1371/journal.pdig.0001171)
Supplement: S1 Table — (DOCX) [file pdig.0001171.s005.docx]

S3 Table. Considered candidate predictors of Dengue Shock Syndrome (DSS) and the combined endpoint of moderate plasma leakage and/or DSS in the Vietnamese dataset. Predictors included in each analysis are denoted by +.

| **No.** | **Variable** | **Meta-analysis^10^** | **Expert opinion*** | **Candidate predictors** | **Lasso selection for DSS** | **Lasso selection for the combine endpoint** |
| --- | --- | --- | --- | --- | --- | --- |
| 1 | Age | + | + | + | - | - |
| 2 | Sex = male | - | - | - | - | - |
| 3 | Overweight | - | + | + | - | - |
| 4 | Headache | - | - | - | - | - |
| 5 | Anorexia | - | - | - | - | - |
| 6 | Nausea | - | - | - | - | - |
| 7 | Vomiting | + | + | + | - | + |
| 8 | Abdominal pain | + | + | + | - | + |
| 9 | Skin haemorrhage | - | + | + | - | - |
| 10 | Presence of Rash | - | - | - | - | - |
| 11 | Mucosal bleeding | + | + | + | - | + |
| 12 | Haemoglobin | - | + | - (High correlation with haematocrit) | - | - |
| 13 | Haematocrit | - | + | +* | + | -* |
| 14 | Platelet count | + | + | + | + | + |
| 15 | White blood cell count | - | + | + | - | + |
| 16 | Neutrophil count | - | + | - (High correlation with white blood cell count) | - | - |
| 17 | Lymphocyte counts | - | + | + | + | + |
| 18 | ALT | + | + | - (High correlation with AST) | - | - |
| 19 | AST | + | + | + | + | + |
| 20 | Serum albumin | + | + | + | - | + |
| 21 | Serotypes | + (DENV2) | - | - (no POCT availability) | - | - |
| 22 | Immune status | + | + | - (no POCT availability) | - | - |

*Haematocrit was not considered among the candidate predictors of the combined endpoints because it was part of the outcome definition; AST: aspartate aminotransferase; ALT alanine aminotransferase; DENV: dengue virus; POCT: point of care tests.
