# Supplementary material for: Early individualized risk prediction using clinical data for children during the febrile phase of dengue in outpatient settings in Vietnam and Thailand
Source: PLOS Digit Health. 2026 Feb 9;5(2):e0001171. doi: 10.1371/journal.pdig.0001171 (PMC12885294; doi:10.1371/journal.pdig.0001171)
Supplement: S2 Table — (DOCX) [file pdig.0001171.s006.docx]

S5 Table. Top 10 model with most selected frequencies by a 1,000-bootstrap resampling technique.

| **Rank** | **Set of predictors included in model development by lasso selection and bootstrapping** | **Inclusion percent** |
| --- | --- | --- |
| 1 | PLT, AST, LC, HCT | 18 |
| 2 | PLT, AST, LC | 10.5 |
| 3 | PLT, AST, HCT | 7.9 |
| 4 | PLT, AST | 4.5 |
| 5 | PLT, AST, LC, HCT, Vomiting | 3.5 |
| 6 | PLT, AST, LC, HCT, Obese | 3.2 |
| 7 | PLT, AST, LC, HCT, WBC | 3.2 |
| 8 | PLT, AST, LC, Obese | 3 |
| 9 | PLT, AST, HCT, Vomiting | 2.5 |
| 10 | PLT, AST, LC, HCT, Abdominal paint or tenderness | 2.3 |
