# Supplementary material for: Early individualized risk prediction using clinical data for children during the febrile phase of dengue in outpatient settings in Vietnam and Thailand
Source: PLOS Digit Health. 2026 Feb 9;5(2):e0001171. doi: 10.1371/journal.pdig.0001171 (PMC12885294; doi:10.1371/journal.pdig.0001171)
Supplement: S4 Table — (DOCX) [file pdig.0001171.s008.docx]

S6 Table. Global model, model selected by lasso selection with the minimum lambda and bootstraps-derived quantities for assessing model's uncertainty for a combined endpoint of moderate plasma leakage or DSS.

|  | **Global model** | |  | **Selected models** | |  |  |  |  |  |
| --- | --- | --- | --- | --- | --- | --- | --- | --- | --- | --- |
| **Predictors** | **Coefficients** | **SE** | **Bootstrap inclusion frequency (%)** | **Coefficients** | **SE** | **RMSD ratio** | **Relative conditional bias (%)** | **Bootstrap median** | **Bootstrap 2.5^th^ percentile** | **Bootstrap 97.5^th^ percentile** |
| Intercept | 3.791 | 0.985 | 100 | 3.824 | 0.984 | 2.424 | -57.522 | 1.627 | -0.235 | 3.512 |
| PLT | -0.01 | 0.001 | 100 | -0.01 | 0.001 | 2.854 | -36.763 | -0.006 | -0.009 | -0.004 |
| LC | -0.902 | 0.13 | 100 | -0.898 | 0.129 | 3.287 | -46.177 | -0.483 | -0.682 | -0.315 |
| AST | 0.004 | 0.001 | 100 | 0.004 | 0.001 | 1.218 | -11.34 | 0.004 | 0.002 | 0.006 |
| ALB | -0.09 | 0.022 | 99.2 | -0.091 | 0.022 | 1.834 | -36.819 | -0.056 | -0.099 | -0.011 |
| WBC | 0.168 | 0.028 | 98.4 | 0.168 | 0.028 | 3.872 | -61.361 | 0.064 | 0.008 | 0.118 |
| Vomiting | 0.249 | 0.137 | 87.8 | 0 | 0 | 1.076 | -24.548 | 0.157 | 0 | 0.413 |
| Mucosal bleeding | 0.625 | 0.305 | 73.3 | 0 | 0 | 1.464 | -46.444 | 0.204 | 0 | 0.78 |
| Abdominal pain or tenderness | 0.415 | 0.352 | 61.8 | 0 | 0 | 0.917 | -18.552 | 0.108 | 0 | 0.809 |
| Age | -0.038 | 0.023 | 29.7 | -0.039 | 0.023 | 1.484 | -61.618 | 0 | -0.032 | 0 |
| Skin bleeding | -0.112 | 0.219 | 12.3 | 0 | 0 | 0.557 | -123.046 | 0 | -0.057 | 0.104 |
| Obese | 0.038 | 0.209 | 11.7 | 0 | 0 | 0.252 | -46.719 | 0 | -0.05 | 0.08 |

PLT: platelet count; LC: lymphocyte count; AST: aspartate aminotransaminase; WBC; white blood cell count; ALB: serum albumin
