# Supplementary material for: Early individualized risk prediction using clinical data for children during the febrile phase of dengue in outpatient settings in Vietnam and Thailand
Source: PLOS Digit Health. 2026 Feb 9;5(2):e0001171. doi: 10.1371/journal.pdig.0001171 (PMC12885294; doi:10.1371/journal.pdig.0001171)
Supplement: S5 Table — (DOCX) [file pdig.0001171.s009.docx]

S7 Table. Top 10 model with most selected frequencies by 1,000 bootstrap resampling techniques for a combined endpoint of moderate plasma leakage or DSS.

| **Rank** | **Set of predictors included in model development by lasso selection and bootstrapping** | **Inclusion percent** |
| --- | --- | --- |
| 1 | PLT, LC, AST, ALB, WBC, Vomiting, Mucosal bleeding, and Abdominal pain or tenderness | 23.2 |
| 2 | PLT, LC, AST, ALB, WBC, Vomiting, and Mucosal bleeding | 13.5 |
| 3 | PLT, LC, AST, ALB, WBC, Vomiting, and Abdominal pain or tenderness, Age | 8.4 |
| 4 | PLT, LC, AST, ALB, WBC, Vomiting, Mucosal bleeding, Abdominal pain or tenderness, Age | 7.2 |
| 5 | PLT, LC, AST, ALB, WBC, Vomiting, Abdominal pain or tenderness | 4.8 |
| 6 | PLT, LC, AST, ALB, WBC, Vomiting, Mucosal bleeding, Age | 4.3 |
| 7 | PLT, LC, AST, ALB, WBC, Vomiting | 3.6 |
| 8 | PLT, LC, AST, ALB, WBC, Vomiting, Abdominal pain or tenderness, Age | 2.6 |
| 9 | PLT, LC, AST, ALB, WBC, Mucosal bleeding, Abdominal pain or tenderness | 2.5 |
| 10 | PLT, LC, AST, ALB, WBC, Vomiting, Mucosal bleeding, Abdominal pain or tenderness, Obese | 2.3 |

PLT: platelet count; LC: lymphocyte count; AST: aspartate aminotransaminase; WBC; white blood cell count; ALB: serum albumin
