# Supplementary material for: Early individualized risk prediction using clinical data for children during the febrile phase of dengue in outpatient settings in Vietnam and Thailand
Source: PLOS Digit Health. 2026 Feb 9;5(2):e0001171. doi: 10.1371/journal.pdig.0001171 (PMC12885294; doi:10.1371/journal.pdig.0001171)
Supplement: S6 Table — (DOCX) [file pdig.0001171.s010.docx]

S8 Table. Summary of models developed using multivariable logistic regression with Lasso selection on the training set.

|  | Estimate | Std. Error | z value | Pr(>\|z\|) |
| --- | --- | --- | --- | --- |
| (Intercept) | 3.364204 | 0.949299 | 3.543883 | 0.000394 |
| Vomiting_ey | 0.276364 | 0.135555 | 2.038755 | 0.041475 |
| MucosalBlExamy | 0.569993 | 0.277106 | 2.056948 | 0.039691 |
| AbdoTendery | 0.406123 | 0.347031 | 1.17028 | 0.241888 |
| WBC | 0.172673 | 0.0275 | 6.279109 | 3.41E-10 |
| **PLT** | -0.01001 | 0.001395 | -7.17262 | 7.36E-13 |
| LYMCount | -0.8398 | 0.121962 | -6.88576 | 5.75E-12 |
| ALB | -0.09198 | 0.021912 | -4.19756 | 2.70E-05 |
| AST | 0.004497 | 0.001131 | 3.974965 | 7.04E-05 |
