# Supplementary material for: Early individualized risk prediction using clinical data for children during the febrile phase of dengue in outpatient settings in Vietnam and Thailand
Source: PLOS Digit Health. 2026 Feb 9;5(2):e0001171. doi: 10.1371/journal.pdig.0001171 (PMC12885294; doi:10.1371/journal.pdig.0001171)
Supplement: S7 Table — (DOCX) [file pdig.0001171.s011.docx]

S9 Table. Optimal hyperparameters selected using Bayesian Global Optimisation with Gaussian Processes of models for dengue shock syndrome.

| **Algorithm** | **Hyperparameters** | **Range** | **Selected values** |
| --- | --- | --- | --- |
| **Models for dengue shock syndrome** | | | |
| RF | mtries | 2 to the max number of predictors | 2 |
|  | min_in_leave | 1 to 50 | 50 |
| XGB | number of trees | 10 to 1000 | 326 |
|  | max_depth | 1 to 20 | 1 |
|  | learning rate | 0.0001 to 0.1 | 0.079 |
|  | gamma | 0.0001 to 0.1 | 0.033 |
| SVM | cost | 0.1 to 10 | 5.904 |
|  | gamma | 0.1 to 1 | 0.104 |
| ANN-2 | Hidden layers drop out _1 | 0 to 0.5 | 0.3 |
|  | Hidden layers drop out _2 | 0 to 0.5 | 0.3 |
|  | hidden neuron_1 | 1 to the max number of predictors | 7 |
|  | hidden neuron_2 | 1 to the max number of predictors | 9 |
|  | Learning rate | 0.0001 to 0.01 | 0.0009 |
| **Models for a combined endpoints of moderate plasma leakage or DSS** | | | |
| RF | mtries | 2 to the max number of predictors | 2 |
|  | min_in_leave | 1 to 50 | 49 |
| XGB | number of trees | 10 to 1000 | 648 |
|  | max_depth | 1 to 20 | 1 |
|  | learning rate | 0.0001 to 0.1 | 0.0366 |
|  | gamma | 0.0001 to 0.1 | 0.0983 |
| SVM | cost | 0.1 to 10 | 3.8284 |
|  | gamma | 0.1 to 1 | 0.2229 |
| ANN-2 | Hidden layers drop out _1 | 0 to 0.5 | 0.3 |
|  | Hidden layers drop out _2 | 0 to 0.5 | 0.5 |
|  | hidden neuron_1 | 1 to the max number of predictors | 6 |
|  | hidden neuron_2 | 1 to the max number of predictors | 11 |
|  | Learning rate | 0.0001 to 0.01 | 0.0057 |
