# Supplementary material for: Early individualized risk prediction using clinical data for children during the febrile phase of dengue in outpatient settings in Vietnam and Thailand
Source: PLOS Digit Health. 2026 Feb 9;5(2):e0001171. doi: 10.1371/journal.pdig.0001171 (PMC12885294; doi:10.1371/journal.pdig.0001171)
Supplement: S8 Table — (DOCX) [file pdig.0001171.s012.docx]

S10 Table. Predictive performance of the risk prediction models for the combined endpoint of moderate plasma leakage and/or DSS in a subgroup analysis trained on hospitalised patients in the Vietnamese dataset on internal validation using 10-fold cross validation.

| **Model** | **Brier score (95% CI)** *(the lower, the better)* | **AUROC (95% CI)** | **Calibration intercept (95% CI)** *(the closer to 0, the better)* | **Calibration slope (95% CI)** *(the closer to 1, the better)* | **Accuracy (95% CI)** | **Sensitivity (95% CI)** | **Specificity (95% CI)** | **PPV (95% CI)** | **NPV (95% CI)** |
| --- | --- | --- | --- | --- | --- | --- | --- | --- | --- |
| Logistic regression using WHO warning signs | 0.189 (0.188, 0.189) | 0.61 (0.608, 0.613) | -0.002 (-0.003, -0.001) | 0.893 (0.852, 0.934) | 0.62 (0.617, 0.623) | 0.605 (0.599, 0.612) | 0.626 (0.62, 0.632) | 0.403 (0.4, 0.406) | 0.798 (0.796, 0.8) |
| Logistic regression with variables selected by lasso selection | 0.194 (0.194, 0.194) | 0.648 (0.647, 0.65) | 0 (-0.001, 0.001) | 0.903 (0.892, 0.914) | 0.644 (0.642, 0.647) | 0.64 (0.634, 0.646) | 0.646 (0.641, 0.652) | 0.428 (0.425, 0.431) | 0.817 (0.815, 0.819) |
| Random forest | 0.197 (0.197, 0.197) | 0.624 (0.621, 0.626) | 0 (-0.002, 0.001) | 1.12 (1.098, 1.142) | 0.624 (0.62, 0.628) | 0.616 (0.609, 0.623) | 0.628 (0.62, 0.635) | 0.406 (0.403, 0.41) | 0.803 (0.801, 0.805) |
| Extreme gradient boosted tree | 0.197 (0.196, 0.197) | 0.633 (0.631, 0.636) | 0 (-0.002, 0.001) | 1.127 (1.104, 1.15) | 0.646 (0.643, 0.65) | 0.611 (0.604, 0.618) | 0.66 (0.654, 0.667) | 0.428 (0.424, 0.431) | 0.808 (0.807, 0.81) |
| Support vector machine | 0.201 (0.201, 0.202) | 0.586 (0.581, 0.591) | -0.002 (-0.005, 0) | 1.032 (0.936, 1.128) | 0.601 (0.597, 0.606) | 0.605 (0.598, 0.613) | 0.6 (0.592, 0.607) | 0.384 (0.38, 0.388) | 0.791 (0.788, 0.794) |
| Artificial neural network:2 hidden layers | 0.197 (0.197, 0.198) | 0.633 (0.63, 0.636) | -0.008 (-0.011, -0.006) | 1.384 (1.32, 1.447) | 0.631 (0.626, 0.635) | 0.627 (0.621, 0.634) | 0.632 (0.624, 0.64) | 0.412 (0.408, 0.416) | 0.808 (0.806, 0.809) |
