# Supplementary material for: Early individualized risk prediction using clinical data for children during the febrile phase of dengue in outpatient settings in Vietnam and Thailand
Source: PLOS Digit Health. 2026 Feb 9;5(2):e0001171. doi: 10.1371/journal.pdig.0001171 (PMC12885294; doi:10.1371/journal.pdig.0001171)
Supplement: S10 Table — (DOCX) [file pdig.0001171.s014.docx]

S12 Table. Lower and upper bounds of hyperparameters used in each machine learning algorithms.

| **Algorithm** | **Hyperparameters** | **Range** |
| --- | --- | --- |
| Random forest | mtries | 2 to the maximum number of predictors |
|  | min_in_leave | 1 to 50 |
| XGB | number of trees | 10 to 1000 |
|  | max_depth | 1 to 20 |
|  | learning rate | 0.0001 to 0.1 |
|  | gamma | 0.0001 to 0.1 |
| SVM | cost | 0.01 to 10 |
|  | gamma | 0.01 to 1 |
| ANN-1 | epochs | 100 to 1000 |
|  | hidden neuron_1 | 1 to the max number of predictors |
|  | Learning rate | 0.0001 to 0.01 |
|  | hidden drop in hidden layer 1 | 0 to 0.5 |
|  | hidden drop in hidden layer 2 | 0 to 0.5 |
| ANN-2 | hidden neuron_1 | 1 to the max number of predictors |
|  | hidden neuron_2 | 1 to the max number of predictors |
|  | Learning rate | 0.0001 to 0.01 |
|  | hidden drop in hidden layer 1 | 0 to 0.5 |
|  | hidden drop in hidden layer 2 | 0 to 0.5 |
| ANN-3 | hidden neuron_1 | 1 to the max number of predictors |
|  | hidden neuron_2 | 1 to the max number of predictors |
|  | hidden neuron_3 | 1 to the max number of predictors |
|  | Learning rate | 0.0001 to 0.01 |
|  | hidden drop in hidden layer 1 | 0 to 0.5 |
|  | hidden drop in hidden layer 2 | 0 to 0.5 |

A logistic regression with lasso selection (LR), random forest (RF), Extreme gradience boosted tree (XGB), support vector machine (SVM), and artificial neural network with 2 hidden layers (ANN).
