# Supplementary material for: Early individualized risk prediction using clinical data for children during the febrile phase of dengue in outpatient settings in Vietnam and Thailand
Source: PLOS Digit Health. 2026 Feb 9;5(2):e0001171. doi: 10.1371/journal.pdig.0001171 (PMC12885294; doi:10.1371/journal.pdig.0001171)
Supplement: S11 Table — (DOCX) [file pdig.0001171.s015.docx]

# Supplementary Methods

## Data collection process

### Vietnamese dataset (training dataset)

Children aged 1 to 15 years presenting to Ho Chi Minh City outpatient departments during the febrile phase of dengue, without signs of plasma leakage or shock, were recruited for a prospective cohort study between 2010 and 2013. Children with an identified source of infection or who could not be contacted via mobile phone during the follow-up period were excluded. Demographic details, clinical history, and the results of physical examinations, together with blood samples for haematology, biochemistry, and diagnostic laboratory (i.e., non-structural protein 1 (NS1) rapid test (NS1 Ag STRIP, Bio-Rad), and quantitative reverse-transcription polymerase chain reaction (qRT-PCR)) were collected at the time of patient enrolment. All ambulatory patients were followed up by daily phone calls to determine whether they had experienced complications or had been hospitalised since the previous call. Daily phone calls were made until the fever resolved and the patient returned to normal daily activities. An electronic case report form based on the WHO 2009 classification was used for data capture^3^. Laboratory-confirmed dengue was defined as a positive result with either one of the following tests: (1) positive qRT- PCR assay; (2) positive NS1 enzyme-linked immunosorbent assay (ELISA) (Platelia Dengue NS1 Ag ELISA, Bio-Rad); or (3) IgM seroconversion from paired blood samples (Panbio, Brisbane, Australia). We used the complete data available for 2,245 Vietnamese children with laboratory-confirmed dengue.

### Thai dataset (external validation dataset)

External model validation was performed using data from a prospective cohort study conducted independently at QSNICH, Bangkok between 1994 and 2008, which enrolled children during the febrile phase of dengue at outpatient clinics. Paediatricians clinically evaluated the children, and the clinical evaluation results were recorded using case record forms. Blood samples were collected, and demographic details were recorded at enrolment. All laboratory-confirmed dengue participants were admitted to the QSNICH hospital and monitored until 24 hours after defervescence (after two consecutive temperatures below 38° Celsius). The first blood sample was collected within the first 24 hours of admission, and blood samples were drawn daily until discharge or for a maximum of five consecutive days. We used the complete data available for 446 children with laboratory-confirmed dengue.

## Outcome definitions

Dengue shock syndrome (DSS) was defined by the presence of either narrow pulse pressure (≤ 20 mmHg) or hypotension according to normal range by age with signs of poor peripheral perfusion, e.g., cold-clammy skin, increased capillary refill time (> 2 seconds), peripheral cyanosis, or skin mottling.

For the combined endpoint, moderate plasma leakage was defined as evidence of plasma leakage with either a greater than 15% rise in haematocrit during illness from baseline and/or evidence of new pleural effusion and/or ascites on ultrasound or chest x-ray in accordance with recommendations from dengue experts^9^. The haematocrit baseline was measured at enrolment within the first 72 hours of illness onset without any clinical signs of plasma leakage or haemodynamic instability. The second measurement was collected at the time of defervescence.

## Outliers and missing data

Outliers were defined as variable values greater or smaller than three times the interquartile range (< mean - 3 IQR or > mean + 3 IQR). The identified outliers were checked with the data providers, and if these were consistent with the possible values, they were retained. Otherwise, they were discarded. We excluded from the analysis of the Vietnamese dataset the limited number of cases, 51 (2.17%), with missing values in the outcome or candidate predictors. The pattern of missingness is presented in **S4 Fig**. We discarded 79 (15%) cases with missing data in the outcome or candidate predictors from the Thai external validation dataset.

## Machine learning models

RF consists of a decision tree created using bootstrap training data samples and random feature selection in decision trees. XGB classification is an additive model of decision trees estimated by gradient descent. SVM is a machine learning classifier using a hyperplane to classify data points into predicted categories. We used the most common SVM with radial basis function kernel (i.e., the Gaussian kernel). ANN is a machine learning algorithm with multiple layers of nonlinear processing units. Due to the limited size of the dataset and the number of outcome occurrences, we considered constructing up to 3 hidden layers with an adaptive learning rate method and rectifier function.

To avoid overfitting, we used Bayesian Global Optimisation with Gaussian Processes in the rBayesianOptimization package^19^ to optimise the hyperparameters of the machine learning models. In the Bayesian optimisation, logarithmic loss and Gaussian process upper confidence bound were applied as a metric and acquisition function, respectively. The lower and upper bounds of the hyperparameters are given in **S12 Table**. The machine learning models were calibrated using Platt’s calibration with 10-fold stratification by the outcome.
