# Supplementary material for: Early individualized risk prediction using clinical data for children during the febrile phase of dengue in outpatient settings in Vietnam and Thailand
Source: PLOS Digit Health. 2026 Feb 9;5(2):e0001171. doi: 10.1371/journal.pdig.0001171 (PMC12885294; doi:10.1371/journal.pdig.0001171)
Supplement: S12 Table — (DOCX) [file pdig.0001171.s016.docx]

This study is a valuable and potentially publishable contribution, particularly in improving early risk stratification in outpatient dengue care. The focus on outpatient populations addresses an important gap in current clinical management, and the findings could support more efficient use of hospital resources in dengue-endemic settings.

- The manuscript lacks sufficient background on the prevalence and burden of dengue in Vietnam and Thailand. Including recent epidemiological data would strengthen the rationale for the study and highlight its public health significance.
- Existing prediction models for dengue severity should be referenced more thoroughly. Some important models are either not cited or not adequately discussed.
- The discussion section should be strengthened with a more detailed comparison between the current model and existing models in terms of design, predictor variables, performance metrics, and clinical applicability.
- It is unclear how secondary dengue infections were measured. The methods should clearly describe how this variable was defined and identified.
- Sentences should begin with words rather than numerals. This applies throughout the manuscript and will improve readability and professionalism.
