# Supplementary material for: Early individualized risk prediction using clinical data for children during the febrile phase of dengue in outpatient settings in Vietnam and Thailand
Source: PLOS Digit Health. 2026 Feb 9;5(2):e0001171. doi: 10.1371/journal.pdig.0001171 (PMC12885294; doi:10.1371/journal.pdig.0001171)
Supplement: S13 Table — (DOCX) [file pdig.0001171.s017.docx]

S13 Table. Dengue shock syndrome predictive performance of the logistic regression with lasso selection and extreme gradient boosted tree risk prediction models trained on the Vietnamese dataset, using different cost values of a false negative results. CI: Confidence interval

| **Cost** | **Accuracy (95% CI)** | **Sensitivity (95% CI)** | **Specificity (95% CI)** | **PPV (95% CI)** | **NPV (95% CI)** |
| --- | --- | --- | --- | --- | --- |
| **Logistic regression with variables selected by lasso selection** | | | | | |
| Baseline | 0.737 (0.732, 0.743) | 0.784 (0.777, 0.79) | 0.735 (0.729, 0.741) | 0.14 (0.138, 0.143) | 0.985 (0.985, 0.986) |
| 2 | 0.878 (0.874, 0.881) | 0.51 (0.499, 0.521) | 0.896 (0.892, 0.901) | 0.233 (0.226, 0.241) | 0.973 (0.972, 0.973) |
| 5 | 0.827 (0.821, 0.832) | 0.632 (0.622, 0.641) | 0.836 (0.83, 0.843) | 0.184 (0.179, 0.19) | 0.978 (0.978, 0.978) |
| 10 | 0.777 (0.773, 0.782) | 0.725 (0.718, 0.732) | 0.78 (0.775, 0.785) | 0.158 (0.154, 0.161) | 0.982 (0.982, 0.983) |
| 20 | 0.736 (0.73, 0.742) | 0.786 (0.779, 0.792) | 0.733 (0.727, 0.74) | 0.14 (0.137, 0.143) | 0.985 (0.985, 0.986) |
| 50 | 0.666 (0.661, 0.671) | 0.863 (0.858, 0.868) | 0.656 (0.651, 0.662) | 0.122 (0.121, 0.124) | 0.989 (0.989, 0.99) |
| 100 | 0.624 (0.619, 0.629) | 0.901 (0.897, 0.905) | 0.610 (0.604, 0.615) | 0.114 (0.113, 0.116) | 0.992 (0.991, 0.992) |
| **Extreme gradient boosted tree (XGB)** | | | | | |
| Baseline | 0.747 (0.741, 0.754) | 0.752 (0.744, 0.759) | 0.747 (0.74, 0.755) | 0.145 (0.142, 0.148) | 0.983 (0.983, 0.984) |
| 2 | 0.875 (0.871, 0.879) | 0.518 (0.507, 0.528) | 0.893 (0.889, 0.898) | 0.228 (0.219, 0.237) | 0.973 (0.973, 0.974) |
| 5 | 0.827 (0.824, 0.831) | 0.627 (0.619, 0.634) | 0.838 (0.834, 0.841) | 0.18 (0.175, 0.185) | 0.978 (0.977, 0.978) |
| 10 | 0.787 (0.783, 0.792) | 0.695 (0.689, 0.701) | 0.792 (0.787, 0.797) | 0.16 (0.156, 0.165) | 0.981 (0.98, 0.981) |
| 20 | 0.745 (0.738, 0.752) | 0.754 (0.746, 0.762) | 0.745 (0.737, 0.752) | 0.144 (0.141, 0.147) | 0.983 (0.983, 0.984) |
| 50 | 0.664 (0.657, 0.671) | 0.841 (0.835, 0.848) | 0.655 (0.647, 0.663) | 0.122 (0.119, 0.125) | 0.988 (0.987, 0.988) |
| 100 | 0.592 (0.582, 0.601) | 0.903 (0.897, 0.909) | 0.576 (0.566, 0.586) | 0.105 (0.103, 0.108) | 0.992 (0.991, 0.992) |
