# Supplementary material for: Early individualized risk prediction using clinical data for children during the febrile phase of dengue in outpatient settings in Vietnam and Thailand
Source: PLOS Digit Health. 2026 Feb 9;5(2):e0001171. doi: 10.1371/journal.pdig.0001171 (PMC12885294; doi:10.1371/journal.pdig.0001171)
Supplement: Attachment — Submitted filename: PDIG-D-25-00314_point_by_point_response_final2.docx [file pdig.0001171.s021.docx]

### **Point-by-point response to reviewers**

### **Early individualised risk prediction using clinical data for children during the febrile phase of dengue in outpatient settings in Vietnam and Thailand**

Sorawat Sangkaew, Bethan Cracknell Daniels, Damien K Ming, Bernard Hernandez, Pau Herrero, Piyarat Suntarattiwong, Siripen Kalayanarooj, Anon Srikiatkhachorn, Alan L. Rothman, Darunee Buddhari, Nguyen Lam Vuong, Phung Khanh Lam, Minh Tuan Nguyen, Bridget Wills, Cameron Simmons, Christl A. Donnelly, Sophie Yacoub, Alison Holmes, Ilaria Dorigatti

**Editor**

**1.1 Dataset split and cross-country generalizability: Both Reviewer 1 and Reviewer 3 ask why the Vietnamese cohort was used for training and the Thai cohort for external validation. Either add the reciprocal analysis (train on Thailand, validate on Vietnam) or supply a clear methodological justification (e.g., events-per-variable constraints, class imbalance). If you run the reciprocal analysis, report AUROC, calibration intercept and slope, and comment on any differences in predictor selection.**

Thank you for raising this important point. We chose the Vietnamese cohort for model development and the Thai cohort for external validation primarily due to sample size and event-per-variable (EPV) considerations. The Thai dataset contained only 28 DSS cases and 184 cases of plasma leakage (as compared to 107 DSS and 219 cases of plasma leakage in Vietnam), which is insufficient for robust model development. Established methodological guidance recommends a minimum of 10–20 events per variable to avoid overfitting and to ensure stable predictor selection. Developing models on the smaller Thai dataset would therefore risk poor calibration and limited generalisability.

This is especially relevant in the light of the cross-validation and jackknife resampling procedure implemented, which would inevitably generate several folds with zero outcome events, which would undermine model stability and validity. In contrast, the larger Vietnamese dataset provided an adequate number of events to support reliable model development and allowed for proper internal validation.

For these reasons, we did not perform a reciprocal analysis. We have clarified this rationale in the revised Methods and Discussion, emphasising that the decision was based on methodological best practices to ensure robust model development and fair external validation.

Summary of changes

- In lines 340 – 342, we added the method section “as the Thai dataset contained too few outcome events for both endpoints to support robust model development without risking overfitting and unstable estimates.”
- In lines 306- 307, “Reciprocal validation using the Thai dataset was not feasible because the limited number of outcome events would not have supported robust model development.”

**1.2 Performance in the Thai cohort and triage implications
+ Expand the Results/Discussion to explain why most models underestimated risk in Thailand while the ANN performed acceptably.
+ Quantify, in practical terms, how many Thai children would avoid unnecessary admission if the optimal Lasso model were applied, and how many at-risk cases it would still capture.**

Thank you for this valuable comment. In the Thai cohort, most models tended to underestimate risk compared with Vietnam, likely reflecting later presentation and differences in baseline case-mix. While the ANN model achieved reasonable discrimination, its calibration was suboptimal (calibration slope 1.742 [95% CI: 1.598–1.886] for DSS; 1.31 [95% CI: 1.264–1.355] for plasma leakage), limiting its practical use. From both an intuitive and clinical perspective, the logistic regression with Lasso selection provided the most balanced and interpretable performance, with good discrimination (AUROC 0.789 [95% CI: 0.787–0.791] for DSS; 0.741 [95% CI: 0.740–0.742] for plasma leakage), acceptable calibration, and comparable sensitivity and specificity.

Among machine-learning methods, extreme gradient boosting (XGBoost) showed the most optimal performance, achieving the highest AUROC (0.785 [95% CI: 0.783–0.787] for DSS; 0.745 [95% CI: 0.744–0.747] for plasma leakage) with good calibration. We have clarified this in the revised Discussion, highlighting that while ANN was flexible, logistic regression with Lasso selection and XGBoost provided more robust and practically applicable performance for clinical use.

In external validation on the Thai cohort, the artificial neural network achieved the highest AUROC for DSS (0.690), but calibration remained imperfect (intercept 0.331, slope 0.912), consistent with the pattern observed in internal validation. By comparison, logistic regression with Lasso selection (AUROC 0.677, intercept 0.188, slope 0.531) and XGBoost (AUROC 0.679, intercept 0.248, slope 0.544) showed slightly lower discrimination but more stable calibration.

When comparing the WHO warning signs with the Lasso logistic regression model, clear differences in triage implications emerged. For DSS, the WHO warning signs identified almost all at-risk cases, missing only 6% (28 cases out of 445), but at the expense of a very high false positive rate, with nearly half of children (209 cases out of 445) unnecessarily classified as requiring admission. By contrast, the Lasso model substantially reduced false positives to 13% (56 cases out of 445), but this came with a much higher false negative rate, missing 26% (116 cases out of 445) of children with DSS. A similar pattern was observed for plasma leakage: the WHO warning signs missed almost no cases (1 case out of 445) but produced an extremely high false positive rate of 76% (338 cases out of 445), whereas the Lasso model halved the false positive rate to 41% (182 cases out of 445) but missed slightly more true cases, with 2% (7 cases out of 445) not captured.

These findings highlight a clear trade-off in the Thai setting: the Lasso model improves efficiency by reducing unnecessary admissions, but at the cost of missing more at-risk children, while the WHO warning signs provide a safer coverage at the expense of higher unnecessary admissions.

| Dataset | Model | Accuracy | Sensitivity | Specificity | TP (%) | TN (%) | FP (%) | FN (%) |
| --- | --- | --- | --- | --- | --- | --- | --- | --- |
| DSS | | | | | | | | |
| Vietnamese | warning signs | 0.52 | 0.64 | 0.51 | 68(0.03) | 1069(0.49) | 1015(0.46) | 39(0.02) |
|  | DSS model | 0.74 | 0.71 | 0.74 | 76(0.03) | 1546(0.71) | 538(0.25) | 31(0.01) |
|  | DSS model with DOI | 0.74 | 0.7 | 0.74 | 75(0.03) | 1551(0.71) | 533(0.24) | 32(0.01) |
| Thai | warning signs | 0.47 | 0.85 | 0.2 | 156(0.35) | 52(0.12) | 209(0.47) | 28(0.06) |
|  | DSS model | 0.61 | 0.37 | 0.79 | 68(0.15) | 205(0.46) | 56(0.13) | 116(0.26) |
|  | DSS model with DOI | 0.61 | 0.39 | 0.76 | 72(0.16) | 199(0.45) | 62(0.14) | 112(0.25) |
| Plasma leakage | | | | | | | | |
| Vietnamese | warning signs | 0.53 | 0.6 | 0.52 | 176(0.08) | 993(0.45) | 907(0.41) | 115(0.05) |
|  | Plasma leakage model | 0.69 | 0.68 | 0.69 | 198(0.09) | 1309(0.6) | 591(0.27) | 93(0.04) |
|  | Plasma leakage with DOI | 0.68 | 0.68 | 0.69 | 197(0.09) | 1303(0.59) | 597(0.27) | 94(0.04) |
| Thai | warning signs | 0.24 | 0.96 | 0.19 | 27(0.06) | 79(0.18) | 338(0.76) | 1(0) |
|  | Plasma leakage model | 0.58 | 0.75 | 0.56 | 21(0.05) | 235(0.53) | 182(0.41) | 7(0.02) |
|  | Plasma leakage with DOI | 0.58 | 0.75 | 0.56 | 21(0.05) | 235(0.53) | 182(0.41) | 7(0.02) |

Summary of changes

- We added a paragraph describing the ANN results in more detail compared with other models. “The artificial neural network achieved the highest AUROC for DSS (0.690), but calibration remained imperfect (intercept 0.331, slope 0.912), consistent with the pattern observed in internal validation. By comparison, logistic regression with Lasso selection (AUROC 0.677, intercept 0.188, slope 0.531) and XGBoost (AUROC 0.679, intercept 0.248, slope 0.544) showed slightly lower discrimination but more stable calibration (Table 3).”
- In lines 219 to 226, we also analysed the triage implications of applying the models in the Thai cohort. “In the Thai cohort, application of the Lasso model reduced false positives compared with WHO warning signs (13% vs. 47% for DSS; 41% vs. 76% for plasma leakage), but at the cost of higher false negatives (26% vs. 6% for DSS; 2% vs. 0% for plasma leakage) (Supplement Table 12).”

**1.3 Time-to-event considerations: Reviewer 1 asks whether the models can predict when DSS will occur. State explicitly that the present work predicts risk rather than timing, then briefly discuss how survival or discrete-time hazard models could be explored in future studies.**

We have now emphasised that our work only predicts risks, not timing, and we have included a discussion of other work predicting time to severe dengue, how this could be done in the future, and the benefits.

Summary of changes:

- The following text has been added to the Discussion, lines 291 to 298: "Additionally, we only considered the risk of severe dengue, whereas previous work has identified clinical fluid accumulation and elevated hematocrit as significant predictors of time taken to severe dengue progression in a hospitalized pediatric population in India [23]. Future work using Cox regression for semi-parametric analysis or discrete-time hazard models that leverage machine learning approaches to predict time to severe dengue progression could provide clinically actionable predictions for optimizing monitoring intensity, timing interventions, and allocating intensive care resources based on individual patient trajectories.”

**1.4 Influence of ‘days to hospital’ and other cohort differences: The Thai participants presented a day later on average. Report whether adding “illness day at presentation” to exploratory models altered discrimination or calibration, and explain your final decision to include or exclude this variable.**

Thank you for this important comment. We re-ran the models including illness day at presentation (DOI) as an additional variable. The results (Supplementary Table 11) showed no improvement in predictive performance. For example, in predicting DSS, the AUROC was 0.789 (95% CI: 0.787–0.791) with the Lasso model and 0.778 (95% CI: 0.704–0.853) when DOI was included. Similarly, calibration slopes and intercepts remained essentially unchanged, and accuracy, sensitivity, and specificity were stable within overlapping confidence intervals. Comparable findings were observed for the combined endpoint of plasma leakage or DSS, where AUROC was 0.741 (95% CI: 0.740–0.742) without DOI and 0.732 (95% CI: 0.693–0.771) with DOI.

Given that all patients were enrolled during the febrile phase—when key clinical manifestations are more relevant than exact illness day—and that DOI did not materially improve discrimination or calibration, we decided to keep the original model.

| **Model** | **Brier score (95% CI)** *(the lower, the better)* | **AUROC (95% CI)** | **Calibration intercept (95% CI)** *(the closer to 0, the better)* | **Calibration slope (95% CI)** *(the closer to 1, the better)* | **Accuracy (95% CI)** | **Sensitivity (95% CI)** | **Specificity (95% CI)** | **PPV (95% CI)** | **NPV (95% CI)** |
| --- | --- | --- | --- | --- | --- | --- | --- | --- | --- |
| **Outcome: dengue shock syndrome** | | | | | | | | | |
| Logistic regression using WHO warning signs | 0.041 (0.040, 0.041) | 0.789 (0.787, 0.791) | -0.003 (-0.005, -0.001) | 1.027 (1.011, 1.043) | 0.756 (0.752, 0.761) | 0.779 (0.773, 0.785) | 0.755 (0.750, 0.76) | 0.154 (0.150, 0.158) | 0.985 (0.985, 0.986) |
| Logistic regression with variables selected by Lasso selection | 0.044 (0.043, 0.044) | 0.789 (0.787, 0.791) | -0.001 (-0.002, 0.001) | 0.942 (0.933, 0.951) | 0.731 (0.725, 0.736) | 0.780 (0.774, 0.787) | 0.728 (0.722, 0.734) | 0.138 (0.135, 0.142) | 0.985 (0.984, 0.985) |
| Logistic regression with variables selected by Lasso selection with DOI | 0.044 (0.037, 0.051) | 0.778 (0.704, 0.853) | 0.002 (-0.072, 0.076) | 0.955 (0.558, 1.351) | 0.703 (0.512, 0.894) | 0.795 (0.551, 1.040) | 0.699 (0.491, 0.907) | 0.124 (0.002, 0.246) | 0.986 (0.970, 1.001) |
| **Outcome: combined endpoint of moderate plasma leakage and/or DSS** | | | | | | | | | |
| Logistic regression using WHO warning signs | 0.101 (0.101, 0.101) | 0.697 (0.696, 0.698) | -0.002 (-0.003, -0.001) | 0.965 (0.957, 0.973) | 0.684 (0.68, 0.689) | 0.661 (0.656, 0.667) | 0.688 (0.682, 0.693) | 0.254 (0.251, 0.257) | 0.930 (0.929, 0.931) |
| Logistic regression using variables selected by Lasso selection | 0.104 (0.104, 0.105) | 0.741 (0.740, 0.742) | 0.001 (0.001, 0.001) | 0.968 (0.962, 0.974) | 0.703 (0.700, 0.707) | 0.706 (0.701, 0.711) | 0.703 (0.698, 0.707) | 0.273 (0.270, 0.275) | 0.94 (0.939, 0.941) |
| Logistic regression with variables selected by Lasso selection with DOI | 0.106 (0.100, 0.112) | 0.732 (0.693, 0.771) | 0.002 (-0.028, 0.033) | 1.007 (0.723, 1.292) | 0.703 (0.555, 0.850) | 0.692 (0.487, 0.898) | 0.704 (0.510, 0.899) | 0.277 (0.167, 0.387) | 0.937 (0.907, 0.968) |

Summary of changes

- In lines 178 to 183, we added the additional analysis results from the model performance including the day of illness (DOI) into the result paragraph
- “Including illness day at presentation (DOI) as an additional predictor did not improve model performance (Supplementary Table 11). For DSS, the AUROC was 0.789 (95% CI: 0.787–0.791) with the Lasso model and 0.778 (95% CI: 0.704–0.853) when DOI was included, with calibration and classification metrics remaining stable. Similar findings were observed for the combined endpoint of plasma leakage or DSS (AUROC 0.741 [95% CI: 0.740–0.742] without DOI vs. 0.732 [95% CI: 0.693–0.771] with DOI).”

**1.5 Additional performance metrics: Reviewers expect to see sensitivity, specificity and 95 % confidence intervals. Add a short Methods paragraph explaining why you led with Brier score, AUROC and calibration, then supply sensitivity and specificity in the main performance text or in a supplementary table.**

Thank you for this valuable comment. As our aim was to evaluate overall model performance and generalisability, we emphasised measures that assess both discrimination and calibration. AUROC provides a threshold-independent measure of discrimination, the Brier score reflects both discrimination and calibration, and the calibration slope/intercept assess agreement between predicted and observed risks. These metrics are particularly appropriate when comparing risk prediction models across populations, as they go beyond binary classification performance.

We agree that sensitivity, specificity, and accuracy are commonly reported indicators, and these results are presented for all models in Table 2. In predicting DSS, the Lasso model achieved a sensitivity of 0.780 (95% CI: 0.774–0.787), specificity of 0.728 (95% CI: 0.722–0.734), and accuracy of 0.731 (95% CI: 0.725–0.736), which were comparable to the WHO warning signs model (sensitivity 0.779 [95% CI: 0.773–0.785], specificity 0.755 [95% CI: 0.750–0.760], accuracy 0.756 [95% CI: 0.752–0.761]). In contrast, for the combined endpoint of moderate plasma leakage or DSS, the Lasso model performed better, with sensitivity 0.706 (95% CI: 0.701–0.711), specificity 0.703 (95% CI: 0.698–0.707), and accuracy 0.703 (95% CI: 0.700–0.707). Similar performance values are observed across the machine-learning models.

Summary of changes

- In lines 53, we added the metrics included sensitivity specificity and accuracy into the abstract
- In lines XXX to XXX, we added the description of the results regarding the sensitivity, specificity NPV and PPV. “Sensitivity, specificity, and accuracy for all models are presented in Table 2. For DSS, the Lasso model achieved a sensitivity of 0.780 (95% CI: 0.774–0.787), specificity of 0.728 (95% CI: 0.722–0.734), and accuracy of 0.731 (95% CI: 0.725–0.736), which were comparable to the WHO warning signs model (sensitivity 0.779 [95% CI: 0.773–0.785], specificity 0.755 [95% CI: 0.750–0.760], accuracy 0.756 [95% CI: 0.752–0.761]). For the combined endpoint of moderate plasma leakage or DSS, the Lasso model performed better, with sensitivity 0.706 (95% CI: 0.701–0.711), specificity 0.703 (95% CI: 0.698–0.707), and accuracy 0.703 (95% CI: 0.700–0.707). Similar patterns were observed across the machine-learning models.”

**1.6 Epidemiological context and prior literature
+ Reviewer 2 requests up-to-date burden estimates. Add recent incidence figures for Vietnam and Thailand to the Introduction to underscore public-health need.
+ Cite and contrast your approach with key outpatient or early-phase models (e.g., Tan et al. 2020; Phakhounthong et al. 2018; Lee et al. 2009). Highlight how your external validation and decision-curve analysis move the field forward.**

Summary of changes:

- In lines 85-89, we have now updated our burden estimates including adding specific figures for Vietnam and Thailand: “Dengue is a mosquito-borne viral disease that heavily burdens public health systems globally, with 3.83 billion (3.45–4.09) people currently living in areas suitable for dengue transmission [1]. In 2024, the number of dengue cases reached the highest level on record, with more than 14,000,000 cases reported globally [2]. Estimates suggest that the disease burden is of 77.8 million cases (95% CI 50.1–101.2 million) each year [3,4]. Approximately 70% of the global dengue cases are estimated to occur in this region [1,5]. The annual incidence of dengue hospitalization in Vietnam and Thailand is estimated at 142 and 136 people per 100,000, respectively [3]. In 2023, it was estimated that the direct medical cost per dengue case increased from $7.51 and $27.82 when treated in outpatient settings compared to $160.09 and $65.84 in outpatient settings for Thailand and Vietnam, respectively [6]. Thus, inpatient care is a primary driver of the global dengue economic burden despite only 7% of cases being treated in inpatient settings [3].”
- In lines 108 to 120, we have also included a deeper characterization of the literature in both the introduction and discussion, highlighting the suggested papers as well as how our approach advances the field. Introduction paragraph: “Clinical data-driven prediction models can estimate individual risk of severe disease, improving patient triage during the early febrile phase of dengue illness. Although several prediction models for dengue severity exist, most rely on hospitalized patient data [13–16], which may create a selection bias toward more severe presentations from the outset. Conversely, risk prediction models using data collected in the early febrile phase from outpatient settings can inform the triage and hospitalization admission of patients. As well as the early identification of high-risk individuals, this also reduces the unnecessary hospitalizations of low-risk patients, which is particularly important in resource-limited settings or during large dengue outbreaks, when healthcare settings can become overwhelmed. We identified five studies that developed risk prediction models using outpatient data [17–21]. All studies showed moderate to high performance on internal training and validation sets; however, no studies included external validation on an independent dataset from a different country, limiting the generalizability of their findings.”
- In lines 253 to 261, we added “Unlike most previous studies [13–16], our models use predictors collected in outpatient settings during the febrile phase, providing earlier estimates of DSS risk to improve triage and patient management. Of the five studies previously predicting risk of severe dengue using outpatient data, two included no validation [18,19], and three performed model validation by splitting their dataset, either temporally or based on missing data [17,20,21]. Thus, by training our model on a Vietnamese dataset and validating it on an external Thai dataset, this is the first study to demonstrate the generalizability of a model trained on outpatient data across independent settings. We found good predictive performance on the external validation dataset (maximum AUROC 0.69) despite differences between the training and validation sets in transmission setting, hospital admission protocol, clinical management, and monitoring frequency.”
- In lines 262 to 267, we added “To our knowledge, ours is also the first study using outpatient data to include a decision-curve analysis, allowing us to evaluate the net clinical benefit of our prediction models compared to hospitalization of all or none of the patients presenting at an outpatient setting, ensuring the models improve patient outcomes. We showed that at low threshold probabilities, machine learning models (ANN) offered the greatest net benefit in predicting DSS.”
- In lines 268 to 275, we also added “Given DSS is rare, we additionally considered a combined endpoint of moderate plasma leakage and/or DSS, increasing the PPV from 0.138 [95%CI 0.135, 0.142] to 0.273 [95%CI 0.270, 0.275] using the Lasso model. This combined endpoint also had a higher PPV compared to a similar study predicting DSS (PPV 0.10 [95%CI 0.09, 0.12]), reducing unnecessary inpatient care [21]. However, failing to identify individuals progressing to severe dengue poses a greater risk. In a sensitivity analysis, we showed how DSS prevalence and the cost of failing to identify at-risk individuals can be incorporated into the prediction model, tailoring it to individual settings and prioritizing DSS identification.”

**1.7 Definition of secondary infection: Clarify, in the Methods, how primary versus secondary dengue was ascertained (e.g., IgM/IgG ratio, PRNT). This was queried by Reviewer 2.**

Summary of changes:

- We have included this information in lines 149 to 150: “Secondary dengue infection (defined as detecting at least one positive dengue-specific IgG on the febrile and convalescence samples) was associated with DSS and the combined endpoint in both countries, while dengue serotype differences were observed in Vietnam but not in Thailand (Supplementary Tables 1-2).”

**1.8 Temporal validity of older data: The Thai cohort dates to 1994-2008 and the Vietnamese cohort to 2010-2013. Reviewer 3 asks whether rising patient age, obesity prevalence and liver enzyme patterns might limit transportability. Add a closing paragraph in the Discussion on secular trends and the need for prospective validation in contemporary cohorts.**

Thank you for this helpful suggestion in response to reviewer 3. We have followed the editor's suggestion and included this point in our discussion.

Summary of changes:

- In lines 315 to 219, we added the point of the suggestion as “Finally, it will be important to assess how the accuracy obtained in the Thai and Vietnamese cohorts (1994-2008 and 2010-2013, respectively) compare to the current and future risk, given temporal changes in dengue patient characteristics, including demographic shifts toward older patients, increasing obesity rates, and higher baseline liver enzyme levels in the region can affect severity [24–27]. Prospective validation of our models in contemporary cohorts is therefore essential.”

**1.9 Editorial and presentation points
+ Rewrite sentences that begin with numerals; observe journal style for abbreviations and US spelling.
+ Re-label Supplementary Table 1 so that the “Overall” column is unambiguous.
+ Ensure figure legends are fully self-contained and that all axes and units are clear.**

We have updated the text, re-labelled Table S1 and updated figure legends as requested.

**Reviewer #1**

**2.1 The models were trained and tested by the Vietnamese data, which makes sense as the data was richer compared with the Thai data set. I do wonder what the outputs might be if the models were trained and tested independently using the Thai dengue data, whether the same set of variables will be identified as the predictors of severe dengue.**

Thank you for raising this point. Please see the response to the Editor in point 1.

**2.2 The variables considered in the model were those that are supposed to be available as a standard measure in outpatient setting. The findings of this study could be practically useful for patient management. I wonder if it could be demonstrated that based on the optimal model achieved in this study, not all the Thai cohort needing hospitalization? Although my understanding was that only the ANN model was well performed on the external validation. All other models seemed to underestimate the risk of severe dengue among the Thai cases. Can you comment on this?**

Thank you for this insightful comment. Although the ANN model achieved reasonable discrimination, its calibration was suboptimal in internal validation (slope 1.742 [95% CI: 1.598–1.886] for DSS; 1.31 [95% CI: 1.264–1.355] for plasma leakage), limiting its practical use. Logistic regression with Lasso selection provided the most balanced and interpretable performance, with good discrimination (AUROC 0.789 [95% CI: 0.787–0.791] for DSS; 0.741 [95% CI: 0.740–0.742] for plasma leakage), acceptable calibration, and comparable sensitivity and specificity. Among machine-learning methods, XGBoost showed the strongest performance, with AUROCs of 0.785 (95% CI: 0.783–0.787) for DSS and 0.745 (95% CI: 0.744–0.747) for plasma leakage, and good calibration.

In external validation on the Thai cohort, the ANN again achieved the highest AUROC for DSS (0.690) but with imperfect calibration (intercept 0.331, slope 0.912), consistent with the pattern observed in internal validation. By comparison, Lasso regression (AUROC 0.677, intercept 0.188, slope 0.531) and XGBoost (AUROC 0.679, intercept 0.248, slope 0.544) showed slightly lower discrimination but more stable calibration.

Summary of changes

- In the lines 213 to 219, we added a paragraph describing the ANN results in more detail compared with other models. “The artificial neural network achieved the highest AUROC for DSS (0.690), but calibration remained imperfect (intercept 0.331, slope 0.912), consistent with the pattern observed in internal validation. By comparison, logistic regression with Lasso selection (AUROC 0.677, intercept 0.188, slope 0.531) and XGBoost (AUROC 0.679, intercept 0.248, slope 0.544) showed slightly lower discrimination but more stable calibration (Table 3).”
- In the lines 245 to 248, we amended the first paragraph of the Discussion to read: “Internal validation showed that logistic regression with predictors selected by Lasso achieved the most balanced performance, combining good discrimination and calibration. Among machine-learning methods, XGBoost and ANN performed well, with XGBoost showing the most consistent and optimal results, particularly in external validation.”

**2.3 One thing that I would like to see in these predictive models is the sense of time to the DSS from patient arriving at hospital. I am not sure if these algorithms could say more on the likely time of plasma leakage and/or DSS e.g. within 12 hrs, 3 days or 7 days from admission etc. This could make the patient management even more efficient, I think**.

We agree that modelling the timing of DSS or plasma leakage would be highly valuable for clinical management. However, this is beyond the scope of the present study, which aimed to predict the occurrence of these outcomes during the acute illness rather than their precise timing. We have noted this as an important direction for future research.

Summary of changes

- In the lines XXX to XXX, we also added to the Discussion and Limitations: “Future work using Cox regression or discrete-time hazard models, including machine learning approaches, to predict time to severe dengue progression could provide clinically actionable predictions to optimize monitoring intensity, guide timely interventions, and allocate intensive care resources based on individual patient trajectories.”

**2.4 Although both Vietnam and Thailand are low resource settings, the data (Supplementary table 1 and 2) showed a difference of a day to reach the hospital. This factor was somehow not made it in the models which I wonder if it affected the lower risk predicted in the Thai cohort. What do the authors think about this?**

Thank you for this thoughtful comment. We explored the effect of including day of illness (DOI) at presentation by performing a sensitivity analysis.

For the DSS outcome, adding DOI had minimal impact on performance: in the Vietnamese dataset, accuracy remained 0.74, with sensitivity 0.71 vs. 0.70 and specificity 0.74 in both models. In the Thai dataset, accuracy was unchanged at 0.61, with sensitivity 0.37 vs. 0.39 and specificity 0.79 vs. 0.76. Similarly, for plasma leakage, accuracy, sensitivity, and specificity were essentially unchanged when DOI was added (e.g., Vietnamese dataset: accuracy 0.69 vs. 0.68, sensitivity 0.68 vs. 0.68, specificity 0.69 in both models).

We present the model performance with and without DOI in both cohorts for DSS and plasma leakage in Supplementary Table 12.

| Dataset | Model | Accuracy | Sensitivity | Specificity | TP (%) | TN (%) | FP (%) | FN (%) |
| --- | --- | --- | --- | --- | --- | --- | --- | --- |
| DSS | | | | | | | | |
| Vietnamese | warning signs | 0.52 | 0.64 | 0.51 | 68(0.03) | 1069(0.49) | 1015(0.46) | 39(0.02) |
|  | DSS model | 0.74 | 0.71 | 0.74 | 76(0.03) | 1546(0.71) | 538(0.25) | 31(0.01) |
|  | DSS model with DOI | 0.74 | 0.7 | 0.74 | 75(0.03) | 1551(0.71) | 533(0.24) | 32(0.01) |
| Thai | warning signs | 0.47 | 0.85 | 0.2 | 156(0.35) | 52(0.12) | 209(0.47) | 28(0.06) |
|  | DSS model | 0.61 | 0.37 | 0.79 | 68(0.15) | 205(0.46) | 56(0.13) | 116(0.26) |
|  | DSS model with DOI | 0.61 | 0.39 | 0.76 | 72(0.16) | 199(0.45) | 62(0.14) | 112(0.25) |
| Plasma leakage | | | | | | | | |
| Vietnamese | warning signs | 0.53 | 0.6 | 0.52 | 176(0.08) | 993(0.45) | 907(0.41) | 115(0.05) |
|  | Plasma leakage model | 0.69 | 0.68 | 0.69 | 198(0.09) | 1309(0.6) | 591(0.27) | 93(0.04) |
|  | Plasma leakage with DOI | 0.68 | 0.68 | 0.69 | 197(0.09) | 1303(0.59) | 597(0.27) | 94(0.04) |
| Thai | warning signs | 0.24 | 0.96 | 0.19 | 27(0.06) | 79(0.18) | 338(0.76) | 1(0) |
|  | Plasma leakage model | 0.58 | 0.75 | 0.56 | 21(0.05) | 235(0.53) | 182(0.41) | 7(0.02) |
|  | Plasma leakage with DOI | 0.58 | 0.75 | 0.56 | 21(0.05) | 235(0.53) | 182(0.41) | 7(0.02) |

Summary of changes

- In lines 254 to 259, we added the additional analysis results from the model performance including the day of illness (DOI) into the result paragraph
- “Including illness day at presentation (DOI) as an additional predictor did not improve model performance (Supplementary Table 10). For DSS, the AUROC was 0.789 (95% CI: 0.787–0.791) with the Lasso model and 0.778 (95% CI: 0.704–0.853) when DOI was included, with calibration and classification metrics remaining stable. Similar findings were observed for the combined endpoint of plasma leakage or DSS (AUROC 0.741 [95% CI: 0.740–0.742] without DOI vs. 0.732 [95% CI: 0.693–0.771] with DOI).”

**2.5 I am not sure why there was little discussion on sensitivity and specificity of these models, perhaps there is a good explanation. I think people usually expect to learn the performance of predictive models through the accuracy, sensitivity and specificity and even some discussions of false positive and false negative. I don’t see much in this study. It would be helpful to say more on other model performance indicators what they are and why they are more appropriate to look at i.e. Brier score, AUROC, Calibration intercept and slope.**

Thank you for the comment. Please see our response to the Editor’s point 2.

**Reviewer #2**

**3.1 The manuscript lacks sufficient background on the prevalence and burden of dengue in Vietnam and Thailand. Including recent epidemiological data would strengthen the rationale for the study and highlight its public health significance.**

We have now updated the background introduction by including adding specific figures for Vietnam and Thailand

Summary of changes

- In lines 85-89, we have now updated our burden estimates including adding specific figures for Vietnam and Thailand: “Dengue is a mosquito-borne viral disease that heavily burdens public health systems globally, with 3.83 billion (3.45–4.09) people currently living in areas suitable for dengue transmission [1]. In 2024, the number of dengue cases reached the highest level on record, with more than 14,000,000 cases reported globally [2]. Estimates suggest that the disease burden is of 77.8 million cases (95% CI 50.1–101.2 million) each year [3,4]. Approximately 70% of the global dengue cases are estimated to occur in this region [1,5]. The annual incidence of dengue hospitalization in Vietnam and Thailand is estimated at 142 and 136 people per 100,000, respectively [3]. In 2023, it was estimated that the direct medical cost per dengue case increased from $7.51 and $27.82 when treated in outpatient settings compared to $160.09 and $65.84 in outpatient settings for Thailand and Vietnam, respectively [6]. Thus, inpatient care is a primary driver of the global dengue economic burden despite only 7% of cases being treated in inpatient settings [3].”

**3.2 Existing prediction models for dengue severity should be referenced more thoroughly. Some important models are either not cited or not adequately discussed.**

We have included a deeper characterization of the literature in the introduction highlighting how our approach advances the field.

Summary of changes

- In lines 108 to 120, we revised the introduction paragraph: “Clinical data-driven prediction models can estimate individual risk of severe disease, improving patient triage during the early febrile phase of dengue illness. Although several prediction models for dengue severity exist, most rely on hospitalized patient data [13–16], which may create a selection bias toward more severe presentations from the outset. Conversely, risk prediction models using data collected in the early febrile phase from outpatient settings can inform the triage and hospitalization admission of patients. As well as the early identification of high-risk individuals, this also reduces the unnecessary hospitalizations of low-risk patients, which is particularly important in resource-limited settings or during large dengue outbreaks, which can quickly overwhelm healthcare settings. We identified five studies that developed risk production models using outpatient data [17–21]. All studies showed moderate to high performance on internal training and validation sets; however, no studies included external validation on an independent dataset from a different country, limiting the generalizability of their findings.”

**3.3 The discussion section should be strengthened with a more detailed comparison between the current model and existing models in terms of design, predictor variables, performance metrics, and clinical applicability.**

Thank you for this helpful suggestion. We have expanded the Discussion to provide a more detailed comparison between our models and existing studies, addressing design, predictors, performance, and clinical applicability.

Summary of changes

- In lines 252 to 261, we add the paragraph in the discussion section: “Unlike most previous studies [13–16], our models use predictors collected in outpatient settings during the febrile phase, providing earlier estimates of DSS risk to improve triage and patient management. Of the five studies previously predicting risk of severe dengue using outpatient data, two included no validation [18,19], and three performed model validation by splitting their dataset, either temporally or based on missing data [17,20,21]. Thus, by training our model on a Vietnamese dataset and validating it on an external Thai dataset, ours is the first to demonstrate the generalizability of a model trained on outpatient data across independent settings. We found good predictive performance on the external validation dataset (maximum AUROC 0.69) despite differences between the training and validation sets in transmission setting, hospital admission protocol, clinical management, and monitoring frequency.”
- In lines 262 to 267, we add the paragraph in the discussion section: “To our knowledge, ours is also the first study using outpatient data to include a decision-curve analysis, allowing us to evaluate the net clinical benefit of our prediction models compared to hospitalization of all or none of the patients presenting at an outpatient setting, ensuring the models improve patient outcomes. We showed that at low threshold probabilities, machine learning models (ANN) offered the greatest net benefit in predicting DSS.”
- In lines 268 to 275, we add the paragraph in the discussion section: “Given DSS is rare, we additionally considered a combined endpoint of moderate plasma leakage and/or DSS, increasing the PPV from 0.138 [95%CI 0.135, 0.142] to 0.273 [95%CI 0.270, 0.275] using the Lasso model. This combined endpoint also had a higher PPV compared to a similar study predicting DSS (PPV 0.10 [95%CI 0.09, 0.12]), reducing unnecessary inpatient care [21]. However, failing to identify individuals progressing to severe dengue poses a greater risk. In a sensitivity analysis, we showed how DSS prevalence and the cost of failing to identify at-risk individuals can be incorporated into the prediction model, tailoring it to individual settings and prioritizing DSS identification.”

**3.4 It is unclear how secondary dengue infections were measured. The methods should clearly describe how this variable was defined and identified.**

Thank you for this comment. We have clarified that secondary dengue infection was associated with both DSS and the combined endpoint in Vietnam and Thailand, while dengue serotype differences were observed only in Vietnam

Summary of changes:

- In lines 149-150, we revised to “Secondary dengue infection (defined as detecting at least one positive dengue-specific IgG on the febrile and convalescence samples) was associated with DSS and the combined endpoint in both countries, while dengue serotype differences were observed in Vietnam but not in Thailand. (Supplementary Tables 1-2)”

**3.5 Sentences should begin with words rather than numerals. This applies throughout the manuscript and will improve readability and professionalism.**

Thank you. We have updated the manuscript throughout.

**Reviewer #3**

**4.1 Why did you choose Vietnamese dataset for training and Thai dataset for validation?  What will happen if you do the reverse way (Thai dataset for training and Vietnamese dataset for validation)?**

Thank you for raising this point. Please see response to Editor point 1.

**4.2 How do differences in patient’s characteristics of the two data sets affect to the models results?**

Thank you for this question. We visualised the predicted probabilities of DSS and plasma leakage against each predictor, stratified by country and found that the patterns of association were consistent across both datasets.

Summary of changes:

- In lines 223 to 226: When predicted probabilities of DSS and plasma leakage were plotted against each predictor and stratified by country, the patterns of association were consistent across the Vietnamese and Thai datasets, suggesting that differences in baseline patient characteristics did not materially alter predictor–outcome relationships (Supplementary Figure 5 and 6).

**4.3 The data set used in this study was from the past (Thai data was from 1994-2008, Vietnamese data was from 2010-2013).  Clinical characteristics of severe dengue patients have changed over time (recent observational data showed that the average age increases and there are higher proportion of obese patients, higher levels of liver transaminase…). Do you think the trained models using the data set in the past would still work well for data in present? Could you discuss more about this issue in the last paragraph of the discussion?**

We agree this is an important limitation, which we have now discussed.

Summary of changes:

- In lines 315 to 319: “Finally, our Thai and Vietnamese cohorts (1994-2008 and 2010-2013, respectively) may have limited contemporary transportability given temporal changes in dengue patient characteristics, including demographic shifts toward older patients, increasing obesity rates, and higher baseline liver enzyme levels in the region [24–27]. Prospective validation of our models in contemporary cohorts is therefore essential.”

**4.4 Could you review the supplementary Table 1 and reformat it, I think the second column “overall” belongs to the Vietnamese dataset.**

Thank you for spotting this error, we have corrected the table.
