# Supplementary material for: Early individualized risk prediction using clinical data for children during the febrile phase of dengue in outpatient settings in Vietnam and Thailand
Source: PLOS Digit Health. 2026 Feb 9;5(2):e0001171. doi: 10.1371/journal.pdig.0001171 (PMC12885294; doi:10.1371/journal.pdig.0001171)
Supplement: Attachment — Submitted filename: Reviewer comments _Trieu Huynh.docx [file pdig.0001171.s020.docx]

Reviewer’s comments

The manuscript is very well written with careful explaining the rationales to do the study, the methods, results and discussion. The manuscript includes many tables and nice figures which are very informative for the readers to understand the data set and the methods. Rarely research centers in Southeast Asia had a collaboration to get a big data which this study can do. I appreciate the research team and thanks all authors for writing up this paper to provide a potential tool to apply in the future to triage the dengue patients with higher risk of severe disease during the febrile phase. It is very important for countries with high burden of dengue, especially during the dengue season when the healthcare systems are overwhelmed.

However, I have several questions for this paper:

1. Why did you choose Vietnamese dataset for training and Thai dataset for validation? What will happen if you do the reverse way (Thai dataset for training and Vietnamese dataset for validation)?
2. How do differences in patient’s characteristics of the two data sets affect to the models results?
3. The data set used in this study was from the past (Thai data was from 1994-2008, Vietnamese data was from 2010-2013). Clinical characteristics of severe dengue patients have changed over time (recent observational data showed that the average age increases and there are higher proportion of obese patients, higher levels of liver transaminase…). Do you think the trained models using the data set in the past would still work well for data in present? Could you discuss more about this issue in the last paragraph of the discussion?
4. Could you review the supplementary Table 1 and reformat it, I think the second column “overall” belongs to the Vietnamese dataset.
